# A 3,5-dinitro­benzoyl derivative of a stereoisomer of glycerol menthonide

**DOI:** 10.1107/S1600536809020960

**Published:** 2009-06-10

**Authors:** Anthony Kiessling, Charles Campana, Margaret E. Kastner

**Affiliations:** a Department of Chemistry and Physics, Mansfield University, Mansfield, PA 16933, USA; b Senior Scientist, Single CrystalDiffraction, Bruker AXS Inc., 5465 East Cheryl Parkway, Madison, WI 53711-5373, USA; c Department of Chemistry, Bucknell University, Lewisburg, PA 17837, USA

## Abstract

The title compound, [(2*S*,5*R*,6*S*,9*R*)-6-isopropyl-9-methyl-1,4-dioxaspiro­[4.5]dec-2-yl]methyl 3,5-dinitro­benzoate, C_20_H_26_N_2_O_8_, was synthesized as part of a study of three-carbon stereochemical systems. The crystallographic assignment of the absolute stereochemistry is consistent with having started with (−)-menthone, the acetal carbon is *R* and the secondary alcohol is *S*. This brings the dinitro­benzoate into approximately the same plane as the menthyl ring and *anti* to the isopropyl group. Close inter­molecular C=O⋯NO_2_ contacts between neighboring mol­ecules [2.8341 (16) Å] contribute to the packing arrangement. The structure was refined as a pseudo-merohedral twin (monoclinic space group *P*2_1_ emulating the ortho­rhom­bic space group *C*222_1_). Application of the twin law 100, 0
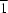
0, 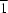
0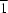 gave a 2:1 ratio of twin moieties [refined BASF value = 0.3790 (7)].

## Related literature

For the synthesis of glycerol menthonide, see: Greenberg (1999 ▶). For the synthesis and NMR spectra of the title compound, see: Kiessling *et al.* (2009 ▶). Glidewell *et al.* (2003 ▶) report a related structure with a very short C=O ⋯ NO_2_ distance. Allen *et al.* (1998 ▶) discuss inter­molecular C=O ⋯ C=O inter­actions. For a description of the Cambridge Crystallographic Database, see: Allen (2002 ▶).
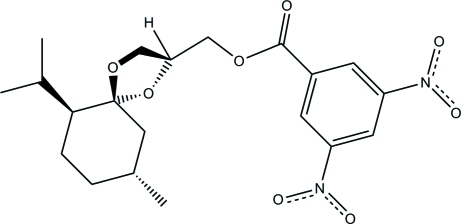


## Experimental

### 

#### Crystal data

C_20_H_26_N_2_O_8_
*M* *_r_* = 422.43
Monoclinic, 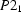

*a* = 9.4396 (5) Å
*b* = 5.8825 (3) Å
*c* = 19.6719 (10) Å
β = 103.923 (3)°
*V* = 1060.26 (9) Å^3^
*Z* = 2
Cu *K*α radiation
μ = 0.87 mm^−1^
*T* = 100 K
0.38 × 0.09 × 0.02 mm

#### Data collection

Bruker SMART APEXII CCD area-detector diffractometer
Absorption correction: multi-scan (*SADABS*; Bruker, 2008 ▶) *T*_min_ = 0.602, *T*_max_ = 0.977
16481 measured reflections
3275 independent reflections
3254 reflections with *I* > 2σ(*I*)
*R*_int_ = 0.025

#### Refinement

*R*[*F* ^2^ > 2σ(*F* ^2^)] = 0.018
*wR*(*F* ^2^) = 0.045
*S* = 1.06
3275 reflections
276 parameters
1 restraint
H-atom parameters constrained
Δρ_max_ = 0.10 e Å^−3^
Δρ_min_ = −0.11 e Å^−3^
Absolute structure: Flack (1983 ▶), 1131 Friedel pairs
Flack parameter: 0.03 (13)

### 

Data collection: *APEX2* (Bruker, 2008 ▶); cell refinement: *SAINT* (Bruker, 2008 ▶); data reduction: *SAINT*; program(s) used to solve structure: *SHELXTL* (Sheldrick, 2008 ▶); program(s) used to refine structure: *SHELXTL*; molecular graphics: *SHELXTL*; software used to prepare material for publication: *SHELXTL*.

## Supplementary Material

Crystal structure: contains datablocks I, global. DOI: 10.1107/S1600536809020960/zl2211sup1.cif

Structure factors: contains datablocks I. DOI: 10.1107/S1600536809020960/zl2211Isup2.hkl

Additional supplementary materials:  crystallographic information; 3D view; checkCIF report

## Figures and Tables

**Fig. 1.**
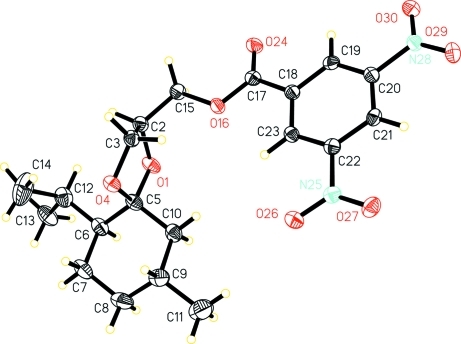
The title compound, C20H26N2O8, showing 50% probability displacement ellipsoids and the atom-numbering scheme.

**Fig. 2.**
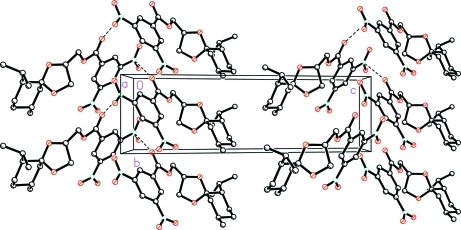
A packing diagram of the title compound. The close intermolecular C═O···NO2 contact is shown by the dotted lines.

## supplementary crystallographic information

### Comment

The title structure was synthesized as part of a study of 3-carbon stereochemical moieties, specifically tri-substituted glycerol. Here menthone serves as a chiral auxiliary, freezing two carbons into a specific stereochemistry and influencing the stereochemistry of the third owing to the steric bulk of the menthone.

The starting material, glycerol menthonide, was originally prepared as an additive to spearmint gum by reaction of menthone with glycerol under acid catalysis. (Greenberg, 1999) No further chemical analysis of the menthonide has been reported in the literature.

Glycerol menthonide exists in as many as six isomers which are difficult to separate. However, conversion of the hydroxy group to an ester by reaction with 4-bromobenzoyl chloride yields a mixture of esters that are separable by flash chromatography.

One stereochemically pure ester was hydrolyzed back to the free alcohol then converted to the 3,5-dinitrobenzoate. The crystallographic assignment of the absolute stereochemistry is consistent with having started with (-)-menthone, and provides the stereochemistry of the acetal carbon and the esterified secondary alcohol of the glycerol chain. Specifically, the acetal carbon, C5, is *R* and the secondary alcohol, C2, is S. This brings the dinitrobenzoate into approximately the same plane as the menthyl ring and anti to the isopropyl group.

There is a close contact between the carbonyl oxygen, O25, and one of the nitro groups on a 2_1_ screw-related molecule, specifically N28 in the molecule at (1 - *x*, -0.5, 2 - *z*). The orientation of the carbonyl group is nearly perpendicular to the plane of the nitro group and the O25 ··· N28 distance is 2.8341 (16) Å. A search of the Cambridge Structural Database (Allen, 2002) for intermolecular C=O ··· NO_2_-benzene groups found 360 observations for C=O ··· NO_2_ distances of 3.07 or less. Of these, only seventeen observations were shorter than that reported here, and each of these had a similar perpendicular orientation. The simplest structure in this set is that of 3-nitrophthalic acid (Glidewell *et al.*, 2003) wherein the C=O ··· NO_2_ distance was reported as 2.807 (2) Å, which the authors attributed to the electrostatic interaction between the partially negative oxygen of the carbonyl and the partially positive nitrogen of the nitro group and analogous to the short intermolecular C=O ··· C=O contacts frequently found between carbonyl groups.

In a study (Allen, *et al.*, 1998) of these intermolecular C=O ··· C=O interactions based on a combination of a detailed analysis of structures from the Cambridge Structural Database as well as *ab initio* molecular-orbital calculations the authors conclude that, although these intermolecular forces are only a fraction of that of hydrogen bonds, they are significant contributors to the stabilization of the solid state structures. It appears a similar argument could be made for C=O ··· NO_2_ interactions.

### Experimental

Details on the synthesis of the title compound and its NMR spectra have been published separately. (Kiessling *et al.*, 2009)

### Refinement

The structure was refined as a pseudo-merohedral twin (Monoclinic space group *P*2_1_ emulating the orthorhombic space group *C*222_1_). Application of the twin law 1 0 0, 0 -1 0, -1 0 -1 gave a 2:1 ratio of twin moieties (refined BASF value 0.3790 (7)).

Hydrogen positions were calculated and refined using a riding model using the following C—H distances: methyne 1.000 Å, methylene 0.990 Å, methyl 0.980Å and aromatic 0.950 Å. The isotropic U values for the H atoms were set at 50% above that of bonded carbon for methyl H atoms and 20% above that of the bonded carbon for all other H atoms.

### Crystal data

**Table:**

C_20_H_26_N_2_O_8_	*F*(000) = 448
*M**_r_* = 422.43	*D*_x_ = 1.323 Mg m^−^^3^
Monoclinic, *P*2_1_	Melting point: 368 K
Hall symbol: P 2yb	Cu *K*α radiation, λ = 1.54178 Å
*a* = 9.4396 (5) Å	Cell parameters from 9947 reflections
*b* = 5.8825 (3) Å	θ = 2.3–68.2°
*c* = 19.6719 (10) Å	µ = 0.87 mm^−^^1^
β = 103.923 (3)°	*T* = 100 K
*V* = 1060.26 (9) Å^3^	Needle, colourless
*Z* = 2	0.38 × 0.09 × 0.02 mm

### Data collection

**Table:**

Bruker SMART APEXII CCD area-detector diffractometer	3275 independent reflections
Radiation source: fine-focus sealed tube	3254 reflections with *I* > 2σ(*I*)
graphite	*R*_int_ = 0.025
φ and ω scans	θ_max_ = 68.2°, θ_min_ = 2.3°
Absorption correction: multi-scan (*SADABS*; Bruker, 2008)	*h* = −11→11
*T*_min_ = 0.602, *T*_max_ = 0.977	*k* = −6→5
16481 measured reflections	*l* = −23→23

### Refinement

**Table:**

Refinement on *F*^2^	Hydrogen site location: inferred from neighbouring sites
Least-squares matrix: full	H-atom parameters constrained
*R*[*F*^2^ > 2σ(*F*^2^)] = 0.018	*w* = 1/[σ^2^(*F*_o_^2^) + (0.0245*P*)^2^ + 0.0832*P*] where *P* = (*F*_o_^2^ + 2*F*_c_^2^)/3
*wR*(*F*^2^) = 0.045	(Δ/σ)_max_ = 0.001
*S* = 1.06	Δρ_max_ = 0.10 e Å^−^^3^
3275 reflections	Δρ_min_ = −0.11 e Å^−^^3^
276 parameters	Extinction correction: *SHELXTL* (Sheldrick, 2008), Fc^*^=kFc[1+0.001xFc^2^λ^3^/sin(2θ)]^-1/4^
1 restraint	Extinction coefficient: 0.0009 (2)
Primary atom site location: structure-invariant direct methods	Absolute structure: Flack (1983), 1131 Friedel pairs
Secondary atom site location: difference Fourier map	Flack parameter: 0.03 (13)

### Special details

**Table:**

Geometry. All e.s.d.'s (except the e.s.d. in the dihedral angle between two l.s. planes) are estimated using the full covariance matrix. The cell e.s.d.'s are taken into account individually in the estimation of e.s.d.'s in distances, angles and torsion angles; correlations between e.s.d.'s in cell parameters are only used when they are defined by crystal symmetry. An approximate (isotropic) treatment of cell e.s.d.'s is used for estimating e.s.d.'s involving l.s. planes.
Refinement. Refinement of *F*^2^ against ALL reflections. The weighted *R*-factor *wR* and goodness of fit *S* are based on *F*^2^, conventional *R*-factors *R* are based on *F*, with *F* set to zero for negative *F*^2^. The threshold expression of *F*^2^ > σ(*F*^2^) is used only for calculating *R*-factors(gt) *etc*. and is not relevant to the choice of reflections for refinement. *R*-factors based on *F*^2^ are statistically about twice as large as those based on *F*, and *R*- factors based on ALL data will be even larger. The structure was refined as a pseudo-merohedral twin (monoclinic space group *P*2_1_ emulating orthorhombic space group *C*222_1_); Twin law 1 0 0 0 - 1 0 - 1 0 - 1; Refined ratio (BASF) 0.3790 (7).Hydrogen positions were calculated and refined using a riding model using the following C—H distances: methyne 1.000 Å, methylene 0.990 Å, methyl 0.980Å and aromatic 0.950 Å. The isotropic U values for the H atoms were set at 50% above that of bonded carbon for methyl H atoms and 20% above that of the bonded carbon for all other H atoms.

### Fractional atomic coordinates and isotropic or equivalent isotropic displacement parameters (Å^2^)

**Table:**

	*x*	*y*	*z*	*U*_iso_*/*U*_eq_	
O1	0.72662 (12)	−0.12929 (19)	0.71101 (5)	0.0258 (3)	
C2	0.81610 (17)	−0.1291 (3)	0.78046 (7)	0.0227 (3)	
H2	0.9171	−0.1783	0.7796	0.027*	
C3	0.81810 (17)	0.1200 (3)	0.80073 (8)	0.0222 (3)	
H3A	0.7329	0.1589	0.8196	0.027*	
H3B	0.9088	0.1591	0.8359	0.027*	
O4	0.81137 (12)	0.23300 (19)	0.73591 (5)	0.0227 (2)	
C5	0.72348 (19)	0.0946 (3)	0.68212 (8)	0.0232 (3)	
C6	0.78923 (18)	0.0943 (3)	0.61799 (8)	0.0266 (4)	
H6	0.7245	−0.0053	0.5824	0.032*	
C7	0.7763 (2)	0.3330 (3)	0.58613 (8)	0.0329 (4)	
H7A	0.8359	0.4400	0.6202	0.039*	
H7B	0.8154	0.3319	0.5437	0.039*	
C8	0.6194 (2)	0.4149 (4)	0.56676 (8)	0.0365 (4)	
H8A	0.5619	0.3166	0.5293	0.044*	
H8B	0.6163	0.5717	0.5482	0.044*	
C9	0.54978 (19)	0.4117 (3)	0.62943 (8)	0.0308 (4)	
H9	0.6041	0.5223	0.6649	0.037*	
C10	0.56642 (18)	0.1768 (3)	0.66303 (8)	0.0270 (4)	
H10A	0.5289	0.1811	0.7059	0.032*	
H10B	0.5063	0.0670	0.6302	0.032*	
C11	0.3900 (2)	0.4850 (4)	0.60923 (11)	0.0461 (5)	
H11A	0.3347	0.3813	0.5736	0.069*	
H11B	0.3831	0.6400	0.5904	0.069*	
H11C	0.3497	0.4809	0.6507	0.069*	
C12	0.9428 (2)	−0.0117 (4)	0.63175 (9)	0.0344 (4)	
H12	0.9459	−0.1414	0.6649	0.041*	
C13	0.9717 (2)	−0.1084 (4)	0.56378 (9)	0.0400 (5)	
H13A	0.9693	0.0152	0.5301	0.060*	
H13B	0.8964	−0.2206	0.5439	0.060*	
H13C	1.0678	−0.1814	0.5740	0.060*	
C14	1.0663 (2)	0.1510 (5)	0.66460 (13)	0.0633 (8)	
H14A	1.1582	0.0664	0.6782	0.095*	
H14B	1.0452	0.2222	0.7061	0.095*	
H14C	1.0748	0.2687	0.6306	0.095*	
C15	0.75391 (17)	−0.2914 (3)	0.82456 (8)	0.0241 (3)	
H15A	0.7341	−0.4403	0.8007	0.029*	
H15B	0.8241	−0.3148	0.8703	0.029*	
O16	0.61928 (12)	−0.19409 (19)	0.83493 (5)	0.0219 (3)	
C17	0.55649 (16)	−0.3039 (3)	0.87874 (7)	0.0204 (3)	
C18	0.43170 (16)	−0.1728 (3)	0.89389 (7)	0.0195 (3)	
C19	0.36462 (15)	−0.2611 (3)	0.94374 (7)	0.0207 (3)	
H19	0.3984	−0.3989	0.9673	0.025*	
C20	0.24814 (16)	−0.1456 (3)	0.95844 (7)	0.0196 (3)	
C21	0.19642 (16)	0.0567 (3)	0.92634 (7)	0.0197 (3)	
H21	0.1167	0.1350	0.9373	0.024*	
C22	0.26696 (16)	0.1397 (3)	0.87730 (8)	0.0203 (3)	
C23	0.38314 (16)	0.0311 (3)	0.86020 (8)	0.0196 (3)	
H23	0.4288	0.0936	0.8264	0.024*	
O24	0.59532 (12)	−0.4860 (2)	0.90476 (6)	0.0267 (3)	
N25	0.21586 (14)	0.3563 (2)	0.84164 (6)	0.0219 (3)	
O26	0.28039 (12)	0.4291 (2)	0.79908 (5)	0.0270 (3)	
O27	0.11232 (11)	0.4517 (2)	0.85721 (6)	0.0275 (3)	
N28	0.17654 (13)	−0.2454 (3)	1.01017 (6)	0.0222 (3)	
O29	0.07569 (12)	−0.1400 (2)	1.02463 (6)	0.0299 (3)	
O30	0.22049 (12)	−0.4300 (2)	1.03556 (6)	0.0271 (3)	

### Atomic displacement parameters (Å^2^)

**Table:**

	*U*^11^	*U*^22^	*U*^33^	*U*^12^	*U*^13^	*U*^23^
O1	0.0394 (6)	0.0178 (6)	0.0215 (5)	−0.0057 (5)	0.0101 (5)	−0.0007 (4)
C2	0.0236 (7)	0.0238 (9)	0.0229 (7)	0.0038 (7)	0.0097 (6)	0.0018 (7)
C3	0.0224 (7)	0.0251 (10)	0.0197 (7)	−0.0025 (7)	0.0061 (6)	−0.0017 (7)
O4	0.0302 (5)	0.0196 (6)	0.0189 (5)	−0.0046 (5)	0.0068 (4)	−0.0012 (5)
C5	0.0325 (8)	0.0158 (9)	0.0218 (7)	−0.0054 (7)	0.0075 (7)	−0.0010 (6)
C6	0.0348 (9)	0.0263 (10)	0.0196 (7)	−0.0092 (8)	0.0083 (7)	−0.0040 (7)
C7	0.0457 (11)	0.0308 (11)	0.0237 (7)	−0.0144 (8)	0.0114 (7)	0.0002 (8)
C8	0.0484 (11)	0.0313 (11)	0.0249 (8)	−0.0089 (9)	−0.0011 (7)	0.0060 (7)
C9	0.0340 (9)	0.0238 (11)	0.0299 (8)	−0.0050 (8)	−0.0013 (7)	0.0004 (7)
C10	0.0279 (8)	0.0261 (10)	0.0266 (7)	−0.0081 (7)	0.0057 (6)	−0.0018 (7)
C11	0.0405 (11)	0.0406 (14)	0.0503 (11)	0.0012 (10)	−0.0023 (9)	0.0048 (10)
C12	0.0388 (10)	0.0405 (12)	0.0272 (8)	−0.0038 (9)	0.0144 (8)	−0.0053 (8)
C13	0.0560 (12)	0.0388 (12)	0.0327 (8)	−0.0020 (10)	0.0253 (9)	0.0000 (8)
C14	0.0360 (11)	0.093 (2)	0.0638 (13)	−0.0085 (12)	0.0177 (10)	−0.0370 (15)
C15	0.0248 (8)	0.0232 (10)	0.0266 (7)	0.0072 (7)	0.0110 (6)	0.0023 (7)
O16	0.0232 (5)	0.0213 (7)	0.0236 (5)	0.0020 (4)	0.0102 (4)	0.0035 (5)
C17	0.0229 (7)	0.0205 (9)	0.0169 (6)	−0.0020 (6)	0.0034 (6)	−0.0026 (6)
C18	0.0196 (7)	0.0211 (9)	0.0172 (6)	−0.0025 (6)	0.0031 (6)	−0.0007 (6)
C19	0.0204 (8)	0.0217 (9)	0.0181 (6)	−0.0002 (7)	0.0007 (6)	0.0010 (6)
C20	0.0200 (7)	0.0222 (9)	0.0163 (6)	−0.0051 (7)	0.0036 (5)	−0.0024 (6)
C21	0.0162 (7)	0.0218 (9)	0.0208 (7)	−0.0004 (6)	0.0037 (5)	−0.0041 (6)
C22	0.0203 (7)	0.0193 (9)	0.0198 (6)	−0.0037 (6)	0.0019 (6)	−0.0015 (6)
C23	0.0206 (7)	0.0202 (9)	0.0184 (7)	−0.0032 (6)	0.0052 (6)	−0.0010 (6)
O24	0.0283 (6)	0.0241 (7)	0.0291 (5)	0.0049 (5)	0.0100 (5)	0.0089 (5)
N25	0.0209 (6)	0.0193 (8)	0.0236 (6)	−0.0027 (6)	0.0017 (5)	−0.0011 (6)
O26	0.0296 (6)	0.0245 (7)	0.0277 (5)	−0.0002 (5)	0.0083 (5)	0.0060 (5)
O27	0.0217 (5)	0.0223 (7)	0.0379 (6)	0.0024 (5)	0.0060 (5)	0.0005 (5)
N28	0.0194 (6)	0.0283 (9)	0.0186 (6)	−0.0040 (6)	0.0043 (5)	−0.0008 (6)
O29	0.0245 (6)	0.0357 (7)	0.0328 (5)	0.0008 (6)	0.0137 (5)	0.0043 (6)
O30	0.0278 (6)	0.0276 (7)	0.0263 (5)	−0.0007 (5)	0.0074 (5)	0.0068 (5)

### Geometric parameters (Å, °)

**Table:**

O1—C2	1.4230 (17)	C12—C13	1.537 (2)
O1—C5	1.432 (2)	C12—H12	1.0000
C2—C15	1.501 (2)	C13—H13A	0.9800
C2—C3	1.518 (2)	C13—H13B	0.9800
C2—H2	1.0000	C13—H13C	0.9800
C3—O4	1.4259 (18)	C14—H14A	0.9800
C3—H3A	0.9900	C14—H14B	0.9800
C3—H3B	0.9900	C14—H14C	0.9800
O4—C5	1.4310 (19)	C15—O16	1.4523 (18)
C5—C10	1.518 (2)	C15—H15A	0.9900
C5—C6	1.534 (2)	C15—H15B	0.9900
C6—C7	1.531 (3)	O16—C17	1.3257 (19)
C6—C12	1.541 (2)	C17—O24	1.206 (2)
C6—H6	1.0000	C17—C18	1.497 (2)
C7—C8	1.517 (3)	C18—C19	1.390 (2)
C7—H7A	0.9900	C18—C23	1.393 (2)
C7—H7B	0.9900	C19—C20	1.381 (2)
C8—C9	1.530 (2)	C19—H19	0.9500
C8—H8A	0.9900	C20—C21	1.380 (2)
C8—H8B	0.9900	C20—N28	1.4726 (19)
C9—C10	1.523 (3)	C21—C22	1.386 (2)
C9—C11	1.526 (3)	C21—H21	0.9500
C9—H9	1.0000	C22—C23	1.379 (2)
C10—H10A	0.9900	C22—N25	1.478 (2)
C10—H10B	0.9900	C23—H23	0.9500
C11—H11A	0.9800	N25—O26	1.2253 (17)
C11—H11B	0.9800	N25—O27	1.2280 (17)
C11—H11C	0.9800	N28—O30	1.2254 (18)
C12—C14	1.526 (3)	N28—O29	1.2256 (18)
			
C2—O1—C5	109.35 (12)	H11A—C11—H11C	109.5
O1—C2—C15	109.28 (13)	H11B—C11—H11C	109.5
O1—C2—C3	102.70 (13)	C14—C12—C13	108.84 (16)
C15—C2—C3	116.37 (13)	C14—C12—C6	114.26 (18)
O1—C2—H2	109.4	C13—C12—C6	110.70 (15)
C15—C2—H2	109.4	C14—C12—H12	107.6
C3—C2—H2	109.4	C13—C12—H12	107.6
O4—C3—C2	102.72 (12)	C6—C12—H12	107.6
O4—C3—H3A	111.2	C12—C13—H13A	109.5
C2—C3—H3A	111.2	C12—C13—H13B	109.5
O4—C3—H3B	111.2	H13A—C13—H13B	109.5
C2—C3—H3B	111.2	C12—C13—H13C	109.5
H3A—C3—H3B	109.1	H13A—C13—H13C	109.5
C3—O4—C5	106.81 (11)	H13B—C13—H13C	109.5
O4—C5—O1	106.07 (11)	C12—C14—H14A	109.5
O4—C5—C10	111.08 (13)	C12—C14—H14B	109.5
O1—C5—C10	108.47 (14)	H14A—C14—H14B	109.5
O4—C5—C6	109.35 (13)	C12—C14—H14C	109.5
O1—C5—C6	110.57 (14)	H14A—C14—H14C	109.5
C10—C5—C6	111.17 (13)	H14B—C14—H14C	109.5
C7—C6—C5	109.12 (14)	O16—C15—C2	107.91 (13)
C7—C6—C12	114.98 (15)	O16—C15—H15A	110.1
C5—C6—C12	113.93 (14)	C2—C15—H15A	110.1
C7—C6—H6	106.0	O16—C15—H15B	110.1
C5—C6—H6	106.0	C2—C15—H15B	110.1
C12—C6—H6	106.0	H15A—C15—H15B	108.4
C8—C7—C6	111.77 (15)	C17—O16—C15	116.25 (13)
C8—C7—H7A	109.3	O24—C17—O16	124.84 (15)
C6—C7—H7A	109.3	O24—C17—C18	123.15 (14)
C8—C7—H7B	109.3	O16—C17—C18	112.00 (14)
C6—C7—H7B	109.3	C19—C18—C23	120.27 (14)
H7A—C7—H7B	107.9	C19—C18—C17	117.49 (15)
C7—C8—C9	112.11 (14)	C23—C18—C17	122.24 (13)
C7—C8—H8A	109.2	C20—C19—C18	118.97 (15)
C9—C8—H8A	109.2	C20—C19—H19	120.5
C7—C8—H8B	109.2	C18—C19—H19	120.5
C9—C8—H8B	109.2	C21—C20—C19	122.72 (14)
H8A—C8—H8B	107.9	C21—C20—N28	119.29 (14)
C10—C9—C11	111.20 (15)	C19—C20—N28	117.99 (15)
C10—C9—C8	109.96 (15)	C20—C21—C22	116.46 (14)
C11—C9—C8	112.06 (15)	C20—C21—H21	121.8
C10—C9—H9	107.8	C22—C21—H21	121.8
C11—C9—H9	107.8	C23—C22—C21	123.38 (15)
C8—C9—H9	107.8	C23—C22—N25	118.12 (13)
C5—C10—C9	112.88 (14)	C21—C22—N25	118.50 (13)
C5—C10—H10A	109.0	C22—C23—C18	118.20 (14)
C9—C10—H10A	109.0	C22—C23—H23	120.9
C5—C10—H10B	109.0	C18—C23—H23	120.9
C9—C10—H10B	109.0	O26—N25—O27	124.54 (14)
H10A—C10—H10B	107.8	O26—N25—C22	117.82 (13)
C9—C11—H11A	109.5	O27—N25—C22	117.64 (12)
C9—C11—H11B	109.5	O30—N28—O29	124.03 (13)
H11A—C11—H11B	109.5	O30—N28—C20	117.93 (13)
C9—C11—H11C	109.5	O29—N28—C20	118.03 (14)
			
C5—O1—C2—C15	145.19 (13)	C5—C6—C12—C13	153.37 (16)
C5—O1—C2—C3	21.05 (15)	O1—C2—C15—O16	−70.24 (16)
O1—C2—C3—O4	−33.31 (15)	C3—C2—C15—O16	45.44 (18)
C15—C2—C3—O4	−152.61 (12)	C2—C15—O16—C17	−173.43 (12)
C2—C3—O4—C5	33.85 (16)	C15—O16—C17—O24	−7.2 (2)
C3—O4—C5—O1	−21.48 (16)	C15—O16—C17—C18	171.59 (12)
C3—O4—C5—C10	96.20 (14)	O24—C17—C18—C19	4.1 (2)
C3—O4—C5—C6	−140.74 (14)	O16—C17—C18—C19	−174.70 (13)
C2—O1—C5—O4	−0.87 (16)	O24—C17—C18—C23	−176.18 (15)
C2—O1—C5—C10	−120.27 (13)	O16—C17—C18—C23	5.01 (19)
C2—O1—C5—C6	117.59 (14)	C23—C18—C19—C20	1.0 (2)
O4—C5—C6—C7	−66.69 (17)	C17—C18—C19—C20	−179.32 (13)
O1—C5—C6—C7	176.87 (13)	C18—C19—C20—C21	−1.0 (2)
C10—C5—C6—C7	56.32 (18)	C18—C19—C20—N28	178.53 (13)
O4—C5—C6—C12	63.30 (19)	C19—C20—C21—C22	0.6 (2)
O1—C5—C6—C12	−53.14 (19)	N28—C20—C21—C22	−178.93 (13)
C10—C5—C6—C12	−173.69 (15)	C20—C21—C22—C23	−0.1 (2)
C5—C6—C7—C8	−56.89 (18)	C20—C21—C22—N25	−179.81 (12)
C12—C6—C7—C8	173.69 (14)	C21—C22—C23—C18	0.2 (2)
C6—C7—C8—C9	56.6 (2)	N25—C22—C23—C18	179.83 (13)
C7—C8—C9—C10	−53.50 (19)	C19—C18—C23—C22	−0.6 (2)
C7—C8—C9—C11	−177.71 (18)	C17—C18—C23—C22	179.73 (14)
O4—C5—C10—C9	65.68 (16)	C23—C22—N25—O26	−0.60 (19)
O1—C5—C10—C9	−178.11 (12)	C21—C22—N25—O26	179.08 (14)
C6—C5—C10—C9	−56.33 (18)	C23—C22—N25—O27	179.99 (13)
C11—C9—C10—C5	178.43 (15)	C21—C22—N25—O27	−0.33 (19)
C8—C9—C10—C5	53.72 (17)	C21—C20—N28—O30	176.82 (13)
C7—C6—C12—C14	43.7 (2)	C19—C20—N28—O30	−2.68 (19)
C5—C6—C12—C14	−83.3 (2)	C21—C20—N28—O29	−2.41 (19)
C7—C6—C12—C13	−79.62 (19)	C19—C20—N28—O29	178.08 (13)
